# Education and Awareness Activities Regarding Hepatitis B and C Among Japanese Dental Health Workers in the Oita Prefecture

**DOI:** 10.7759/cureus.29670

**Published:** 2022-09-27

**Authors:** Yumiko Nagao, Tetsuya Kimura, Kiyohide Tomooka, Haruhiko Wakita

**Affiliations:** 1 Department of Public Health, Graduate School of Medicine, Juntendo University, Tokyo, JPN; 2 Liver Center, Saga University Hospital, Saga, JPN; 3 Dental Health, Oita Dental Association, Oita, JPN

**Keywords:** hepatitis b vaccine, questionnaire, hepatitis c virus, hepatitis b virus, dental healthcare workers, dentist

## Abstract

Objectives: Hepatitis B virus (HBV) and Hepatitis C virus (HCV) infections are known to pose a major threat for dental health workers (DHWs). Previously, we reported that the HBV and HCV infection rates among DHWs in the Oita Prefecture in Japan were higher than those among the general population. The aim of this study was to disseminate knowledge about hepatitis and its prevention among the DHWs.

Materials and Methods: Educational booklets were mailed to 2,197 DHWs working in 487 dental clinics. After anonymously responding to online questions about their experience with HBV and HCV testing, Hepatitis B vaccination, and percutaneous injury, the subjects were required to respond to additional questions about their understanding of hepatitis.

Results: A total of 521 DHWs (205 males and 316 females) responded to the questionnaires. Among them, 61.6% had experienced percutaneous injuries, but only 19.4% were fully aware of how to deal with them before reading the booklet, and also 10.6% had sufficient knowledge about hepatitis. The past Hepatitis B vaccination, past HBV testing rate, and past HCV testing rate were 62.4%, 71.8%, and 43.2%, respectively. The DHWs who are not dentists (n = 293) had significantly lower rates of past testing for their own hepatitis virus, knowledge about treatment of percutaneous injuries, and awareness of hepatitis as compared to dentists (n = 228). After reading the booklet, 99.5% of subjects found the booklet useful and 87.3% said it would influence their future hepatitis testing.

Conclusion: The educational booklet was effective way to increase DHWs' knowledge about Hepatitis B and C and how to manage percutaneous injuries.

## Introduction

According to the WHO, approximately 257 million and 71 million people were infected with Hepatitis B virus (HBV) and Hepatitis C virus (HCV), respectively, worldwide in 2015 [1]. Liver cancer is the sixth most common cancer and the fourth most common cause of cancer-related death in the world, with increasing incidence rates in many countries [2,3]. In 2015, Japan reported an estimated 1.1-1.2 million HBV-infected and 900,000-1.3 million HCV-infected people [4]. Most of the cases of liver cancer in Japan are caused by HBV and HCV infections [5]. Liver cancer is characterized by a high mortality rate in western Japan [6], with approximately 25,000 liver cancer deaths in 2019. In Japan, antiviral therapies such as direct-acting antivirals and nucleic acid analogs have been introduced and mortality rates due to liver cancer have decreased after the comprehensive promotion of measures against viral hepatitis, based on the Basic Law on Hepatitis Measures [7-9].

Dental health workers (DHWs) are at risk for occupational hazards owing to frequent contact with the blood, saliva, and body fluids of patients. In addition to blood-borne pathogens such as HBV, HCV, and human immunodeficiency virus (HIV) [10], dentists need to focus on aerosol infections such as severe acute respiratory syndrome coronavirus 2 (SARS-CoV2) [11]. Contact with pathogens can spread among patients, DHWs, and between patients and DHWs.

In Japan, the revised Medical Service Act ("Act for Partial Revision of the Medical Service Act for the Purpose of Establishing a System for Providing Quality Medical Care") came into effect in April 2007, making it mandatory for all dental clinics to establish medical safety. Medical institutions, regardless of their size, are required by the Medical Service Act to prepare and implement guidelines for ensuring the following: medical safety, systems for nosocomial infection control, systems for pharmaceutical safety, and systems for medical device safety. Furthermore, in 2018, nosocomial infection prevention measures were established as a new facility standard in the basic consultation fee for dentistry.

Knowledge about HBV- and HCV-infected patients and infection control is essential among DHWs. However, in our previous survey comprising Japanese DHWs in 2007, the prevalence of HBV infection was high at 12.1% and the rate of Hepatitis B vaccination rate was low at 48.2% [12]. A questionnaire survey of domestic dental students and DHWs (mainly dentists) in the Japanese Society of Dental Practice Administration indicated insufficient knowledge about nosocomial infection control and viral hepatitis [13,14]. Furthermore, a large-scale study comprising 1,834 members of the Dental National Health Insurance Society in the Oita Prefecture revealed that the rates of HBV and HCV infection were higher among the dental workers when compared to those among general blood donors in Japan [15]. The positivity rates of the Hepatitis B surface antigen (HBsAg), HBsAg antibodies (anti-HBs), and HCV antibodies (anti-HCV) among the DHWs in the Oita Prefecture were 0.6%, 44.1%, and 0.5%, respectively; these rates were found to increase with age and were particularly high (1.7%-2.2%) among those in the 50-70 age group [15].

In the present study, educational booklets on hepatitis were distributed to all DHWs in the Oita Prefecture to promote knowledge about hepatitis and the necessity of hepatitis virus testing. The purpose of this study was to investigate the awareness of hepatitis virus and health management such as testing and prevention before reading the booklet, and to investigate the level of understanding of hepatitis and willingness to undergo testing after reading the booklet, both in a single questionnaire.

## Materials and methods

Educational booklet on Hepatitis C and B for DHWs

An educational booklet titled, "Basics of Hepatitis that Dentists Should Know", was prepared to provide a common curriculum to all subjects. The contents of this educational booklet were as follows: (1) significance of HBV and HCV testing for DHWs; (2) difference between Hepatitis B and C; (3) Hepatitis B vaccination methods; (4) testing methods for HBV and HCV infection; (5) relationship between viral hepatitis and dental diseases, especially oral mucosal diseases such as oral lichen planus; (6) how to deal with patients who are hepatitis carriers; and (7) how to deal with needlestick accidents.

Study design

On June 28, 2021, 2,197 copies of this educational booklet were delivered by mail to 2,197 DHWs working at 487 dental clinics in the Oita Prefecture. The questionnaire was administered online from June 29 to September 30, 2021, and the subjects were requested to complete them anonymously (Table 1). The questions in the online survey are Q1-Q13 shown in Table 1. The number of surveys to the target audience was set to one, but the questions included questions before and after reading the booklet. Q4-Q9 asked about knowledge before reading the booklet, and Q10-Q13 asked about the effects after reading the booklet. Participation in the survey was voluntary; an explanation about the study was provided on the web-based questionnaire response form and completion of the questionnaire constituted consent. The questionnaire was answered once per person using a smartphone, and no reward was given for the answers. The results of the questionnaire were compared between dentists and DHWs other than dentists (e.g., dental hygienists, dental technicians, dental assistants), as well as between DHWs who had experienced percutaneous injuries and those who had not.

**Table 1:** The items and responses in the questionnaire DHWs: Dental healthcare workers; HCV: Hepatitis C virus; HBV: Hepatitis B virus

No.	Questions	Answer items
1	What is your age (years) ?	18-19 / 20-29 / 30-39 / 40-49 / 50-59 / 60-69 / 70-79 / ≥80
2	What is your gender ?	Male / Female
3	Please select your dental occupation.	Dentists / DHWs other than dentists
4	Before reading this booklet, have you ever been tested for HCV?	Yes / No / Not sure
5	Before reading this booklet, have you ever been tested for HBV?	Yes / No / Not sure
6	Before reading this booklet, have you ever been vaccinated against hepatitis B?	Yes / No / Don't know
7	Before reading this booklet, have you ever had an accidental needlestick or cut during dental work?	Yes / No / Don't know
8	Before reading this booklet, did you know how to respond to a needlestick or cut wound development?	Know enough / Know almost enough / Know little / Know nothing at all
9	Before reading this booklet, how much did you know about hepatitis C and hepatitis B?	Know enough / Know almost enough / Know little / Know nothing at all
10	After reading this booklet, do you have a better understanding of HBV and HCV infection than before?	Yes / Somewhat / Not really / No
11	After reading this booklet, did you want to be tested for hepatitis virus infection?	Yes / Somewhat / Not sure / Not much / No
12	After reading this booklet, did you want to recommend people around you (family, acquaintances, patients, etc.) to be tested for hepatitis virus infection?	Yes / Somewhat / Not sure / Not much / No
13	After reading this booklet, please provide any comments or opinions about the booklet (free answer, optional)	

Statistical analysis

Differences between groups were analyzed using the X²-test. All statistical analyses were performed using JMP version 13 (SAS Institute, Inc.). A p-values of < 0.05 indicated statistical significance.

Ethical approval

The study protocol was approved by the Ethics Committee of the Oita Dental Association (approval no. 2) and was performed in accordance with the Declaration of Helsinki.

## Results

Results of the online survey

A total of 521 out of 2,197 DHWs (23.7%) comprising 205 males and 316 females completed the online questionnaire anonymously (Table 2).

**Table 2:** Results of the responses to the questionnaire DHWs: Dental healthcare workers; HCV: Hepatitis C virus; HBV: Hepatitis B virus; NS: Not significant

	Questions	Answer items	Total		Dentists		DHWs other than dentists		p-value
			521		228		293		
1	Age (years)	18–19	0	0.00%	-	-	0	-	<0.0001
		20–29	85	16.30%	0	0.00%	85	29.00%	
		30–39	85	16.30%	24	10.50%	61	20.80%	
		40–49	120	23.00%	46	20.20%	74	25.30%	
		50–59	103	19.80%	50	21.90%	53	18.10%	
		60–69	93	17.90%	75	32.90%	18	6.10%	
		70–79	33	6.30%	31	13.60%	2	0.70%	
		≥80	2	0.40%	2	0.90%	0	0.00%	
2	Gender	Male	205	39.30%	200	87.70%	5	1.70%	<0.0001
		Female	316	60.70%	28	12.30%	288	98.30%	
3	Before reading this booklet, have you ever been tested for HCV?	Yes	225	43.20%	127	55.70%	98	33.40%	<0.0001
		No	214	41.10%	68	29.80%	146	49.80%	
		Not sure	82	15.70%	33	14.50%	49	16.70%	
4	Before reading this booklet, have you ever been tested for HBV?	Yes	374	71.80%	191	83.80%	183	62.50%	<0.0001
		No	108	20.70%	25	11.00%	83	28.30%	
		Not sure	39	7.50%	12	5.30%	27	9.20%	
5	Before reading this booklet, have you ever been vaccinated against hepatitis B?	Yes	325	62.40%	150	65.80%	175	59.70%	NS
		No	154	29.60%	65	28.50%	89	30.40%	
		Don't know	42	8.10%	13	5.70%	29	9.90%	
6	Before reading this booklet, have you ever had an accidental needlestick or cut during dental work?	Yes	321	61.60%	167	73.20%	154	52.60%	<0.0001
		No	186	35.70%	57	25.00%	129	44.00%	
		Don't know	14	2.70%	4	1.80%	10	3.40%	
7	Before reading this booklet, did you know how to respond to a needlestick or cut wound development?	Know enough	101	19.40%	64	28.10%	37	12.60%	<0.0001
		Know almost enough	316	60.70%	140	61.40%	176	60.10%	
		Know little	95	18.20%	23	10.10%	72	24.60%	
		Know nothing at all	9	1.70%	1	0.40%	8	2.70%	
8	Before reading this booklet, how much did you know about hepatitis C and hepatitis B?	Know enough	55	10.60%	39	17.10%	16	5.50%	<0.0001
		Know almost enough	301	57.80%	153	67.10%	148	50.50%	
		Know little	165	31.70%	36	15.80%	129	44.00%	
		Know nothing at all	0	0.00%	0	0.00%	0	0.00%	
9	After reading this booklet, do you have a better understanding of HBV and HCV infection than before?	Yes	365	70.10%	174	76.30%	191	65.20%	0.0227
		Somewhat	153	29.40%	53	23.20%	100	34.10%	
		Not really	3	0.60%	1	0.40%	2	0.70%	
		No	0	0.00%	0	0.00%	0	0.00%	
10	After reading this booklet, did you want to be tested for hepatitis virus infection?	Yes	245	47.00%	104	45.60%	141	48.10%	NS
		Somewhat	210	40.30%	98	43.00%	112	38.20%	
		Not sure	50	9.60%	18	7.90%	32	10.90%	
		Not much	11	2.10%	4	1.80%	7	2.40%	
		No	5	1.00%	4	1.80%	1	0.30%	
11	After reading this booklet, did you want to recommend people around you (family, acquaintances, patients, etc.) to be tested for hepatitis virus infection?	Yes	193	37.00%	100	43.90%	93	31.70%	0.0003
		Somewhat	222	42.60%	101	44.30%	121	41.30%	
		Not sure	82	15.70%	21	9.20%	61	20.80%	
		Not much	14	2.70%	5	2.20%	9	3.10%	
		No	10	1.90%	1	0.40%	9	3.10%	

Among them, the highest proportion of subjects (120 respondents, 23%) belonged to the 40-49 age group and 61.6% experienced needlestick cuts/ wounds. However, only 19.4% knew how to respond to the injury before reading the booklet. Furthermore, 62.4% of the 521 subjects were vaccinated against Hepatitis B, 71.8% were previously tested for HBV, and 43.2% were previously tested for HCV. Only 10.6% of the subjects had sufficient knowledge about Hepatitis B and C before reading the booklet. After reading the booklet, nearly all subjects (99.5%) agreed that the educational booklet increased their understanding of hepatitis. After reading the booklet, 87.3% of the subjects were motivated to get tested for hepatitis virus (those who answered "yes" or "somewhat"), and 79.6% answered that it would influence them to encourage others to get tested for hepatitis virus (those who answered "yes" or "somewhat"). 54 of the 521 respondents expressed their comments and opinions corresponding to Q13 in Table 1. The opinions of the 54 respondents can be roughly categorized as follows: effectiveness of improving knowledge of hepatitis (28 respondents), awareness of the importance of infection control measures (8 respondents), awareness of what to do in case of needlestick accidents (8 respondents), importance of Hepatitis B vaccine (6 respondents), having taken or going to take hepatitis tests after reading the booklet (2 respondents), and awareness of the relationship between oral lichen planus and Hepatitis C (2 respondents).

Differences in the results of the questionnaire based on occupation

As shown in Table 2, significant differences in the following items were observed between the dentists (228 subjects) and DHWs other than dentists (293 subjects): age (P < 0.0001); gender (P < 0.0001); past HCV testing rate (P < 0.0001); past HBV testing rate (P < 0.0001); needlestick and cut experience rate (P < 0.0001); knowledge about how to deal with the needlestick/cut before reading the booklet (P < 0.0001); awareness about Hepatitis B and C before reading the booklet (P < 0.0001); understanding of the booklet (P = 0.0227); and effectiveness of the booklet in encouraging others to get tested for hepatitis virus after reading it (P = 0.0003). The rate of previous hepatitis virus screening, knowledge about needlestick and cut wounds, and awareness of Hepatitis B and C were significantly lower among DHWs other than dentists when compared to those among dentists.

Differences between those who did and did not experience needlestick/cut wounds

Items with significant differences between those who experienced needlestick/cut wounds (n = 321) and those who did not (including unknown; n = 200) were as follows: age (P = 0.0006), gender (P = 0.0002), occupation (P < 0.0001), past HCV testing rate (P = 0.0021), past HBV testing rate (P < 0.0001), and awareness of Hepatitis B and C before reading the booklet (P = 0.0289) (Table 3).

**Table 3:** Comparison between those who experienced needlestick and cut wounds and those who did not DHWs: Dental healthcare workers; HCV: Hepatitis C virus; HBV: Hepatitis B virus; NS: Not significant

	Questions	Answer items	Total		Those who experienced needlestick/cut wounds		Those with no or unknown needlestick/cut experience		p-value
			521		321		200		
1	Age (years)	18–19	0	0.00%	-	-	0	-	0.0006
		20–29	85	16.30%	42	13.10%	43	21.50%	
		30–39	85	16.30%	45	14.00%	40	20.00%	
		40–49	120	23.00%	67	20.90%	53	26.50%	
		50–59	103	19.80%	72	22.40%	31	15.50%	
		60–69	93	17.90%	72	22.40%	21	10.50%	
		70–79	33	6.30%	21	6.50%	12	6.00%	
		≥80	2	0.40%	2	0.60%	0	0.00%	
2	Gender	Male	205	39.30%	147	45.80%	58	29.00%	0.0002
		Female	316	60.70%	174	54.20%	142	71.00%	
3	Please select your dental occupation	Dentists	228	43.80%	167	52.00%	61	30.50%	<0.0001
		DHWsother than dentists	293	56.20%	154	48.00%	139	69.50%	
4	Before reading this booklet, have you ever been tested for HCV?	Yes	225	43.20%	150	46.70%	75	37.50%	0.0021
		No	214	41.10%	113	35.20%	101	50.50%	
		Not sure	82	15.70%	58	18.10%	24	12.00%	
5	Before reading this booklet, have you ever been tested for HBV?	Yes	374	71.80%	254	79.10%	120	60.00%	<0.0001
		No	108	20.70%	48	15.00%	60	30.00%	
		Not sure	39	7.50%	19	5.90%	20	10.00%	
6	Before reading this booklet, have you ever been vaccinated against hepatitis B?	Yes	325	62.40%	206	64.20%	119	59.50%	NS
		No	154	29.60%	93	29.00%	61	30.50%	
		Don't know	42	8.10%	22	6.90%	20	10.00%	
7	Before reading this booklet, did you know how to respond to a needlestick or cut wound development?	Know enough	101	19.40%	67	20.90%	34	17.00%	NS
		Know almost enough	316	60.70%	196	61.10%	120	60.00%	
		Know little	95	18.20%	55	17.10%	40	20.00%	
		Know nothing at all	9	1.70%	3	0.90%	6	3.00%	
8	Before reading this booklet, did you know about hepatitis C and hepatitis B?	Know enough	55	10.60%	37	11.50%	18	9.00%	0.0289
		Know almost enough	301	57.80%	196	61.10%	105	52.50%	
		Know little	165	31.70%	88	27.40%	77	38.50%	
		Know nothing at all	0	0.00%	0	0.00%	0	0.00%	
9	After reading this booklet, do you have a better understanding of HBV and HCV infection than before?	Yes	365	70.10%	219	68.20%	146	73.00%	NS
		Somewhat	153	29.40%	100	31.20%	53	26.50%	
		Not really	3	0.60%	2	0.60%	1	0.50%	
		No	0	0.00%	0	0.00%	0	0.00%	
10	After reading this booklet, did you want to be tested for hepatitis virus infection?	Yes	246	47.20%	146	45.50%	100	50.00%	NS
		Somewhat	210	40.30%	135	42.10%	75	37.50%	
		Not sure	50	9.60%	28	8.70%	22	11.00%	
		Not much	11	2.10%	8	2.50%	3	1.50%	
		No	4	0.80%	4	1.20%		0.00%	
11	After reading this booklet, did you want to recommend people around you (family, acquaintances, patients, etc.) to be tested for hepatitis virus infection?	Yes	197	37.80%	112	34.90%	85	42.50%	NS
		Somewhat	222	42.60%	147	45.80%	75	37.50%	
		Not sure	82	15.70%	51	15.90%	31	15.50%	
		Not much	14	2.70%	5	1.60%	9	4.50%	
		No	6	1.20%	6	1.90%	0	0.00%	

Compared to non-experienced persons, those who had experienced percutaneous injures were significantly older, had a significantly higher percentage of previous HCV and HBV testing, and were significantly more likely to have known about hepatitis B and C before reading the booklet. However, no significant differences in Hepatitis B vaccination history or knowledge about how to respond to needlestick/cut wounds were observed between the two groups.

## Discussion

In the current study, an educational booklet was provided to the DHWs in the Oita Prefecture, following which they were asked to complete a questionnaire comprising of questions about hepatitis. The online survey consisted of content before (e.g., knowledge and healthcare) and after (e.g., understanding and future testing) reading the booklet. The rate of needlestick and cut injuries among the DHWs was 61.6% (n = 321), with dentists being significantly more exposed than DHWs other than dentists (73.2% vs. 52.6%; P < 0.0001). These findings were similar to those reported previously in Japan (70.3% for dentists and 77.2% for dental hygienists) [16]. In Europe, 72% of dentists sustained at least one percutaneous injury while working [17]; in Lithuania, the rate was reported to be 78.5% [18]. In Canada, an average of 1.5 mucosal exposures and three transvascular injuries per gynecologist per year have been reported [19]. The rate of acquiring HBV infection is higher in unvaccinated individuals, ranging from 12% [20] to 60% [21].

The Hepatitis B vaccination rate in this study was 62.4% (n = 325). This rate is an improvement over the rate reported in 2008 (48.2%)[12] but is considerably lower than those reported in other countries, such as Switzerland (94.7%) [22], Iran (94.3%) [23], Brazil (91.2%) [24], and Germany (89.8%) [25].

Before reading the educational booklet, only 19.4% of the subjects knew how to respond to a needlestick/cut wound and 10.6% knew about Hepatitis B and C. The educational booklet helped 99.5% of the subjects to learn about the disease and 87.3% said that it would affect their future hepatitis examinations. This suggests that hepatitis education using the booklet was effective in increasing the knowledge and raising awareness of the disease. However, there were significant differences between dentists and DHWs other than dentists in their understanding of hepatitis and their recommendation of hepatitis to others after reading the educational booklet (corresponding to Q9 and Q11 in Table 2). Dentists were more aware of hepatitis and more likely to recommend hepatitis testing to others than DHWs other than dentists. This may be due to the fact that DHWs other than dentists are less likely to receive training on nosocomial infections. Nakano reported on the current national situation regarding the provision of learning opportunities for graduates by dental hygienist schools [26]. She reported that 48.2% of training sessions were conducted for graduating dental hygienists and dental hygienists in the community, and that 13.5% of these sessions focused on nosocomial infections as the overall topic. In 2020, 90.9% of dental hygienists working in Japan will be employed in dental clinics and 73.4% of dental technicians will be employed in dental laboratories [27]. Considering this, it is necessary to increase the number of training sessions that DHWs other than dentists can attend and to make their attendance at training sessions known.

In addition, it is worthy to note that DHWs are not legally required to be tested for HBV and HCV infection, although the Japanese Society for Infection Prevention and Control clarified vaccine guidelines for healthcare professionals in 2009, and Hepatitis B vaccines are becoming standardized nationwide. They are not legally required to receive the Hepatitis B vaccine despite the high risk of infection. Furthermore, DHWs are expected to acquire basic knowledge about hepatitis through their own efforts; seminars on the main topic hepatitis are rarely held for DHWs in Japan. Hence, opportunities for DHWs, including dentists, to receive the latest information about the disease are limited.

Previously, we demonstrated that dentists in private dental clinics had significantly higher risk scores for infection control and lower knowledge scores for hepatitis than those working in hospitals [14]. Adequate knowledge about Hepatitis B is correlated with safe practices [28,29]. In the current study, the acquisition of knowledge about hepatitis promoted the willingness of the DHWs to be tested for hepatitis virus. Thus, raising awareness and increasing the knowledge about hepatitis among DHWs should not only lead to infection control but also in the control of liver cancer [30]. Systemic efforts must be urgently established to educate all DHWs about hepatitis to encourage them to undergo hepatitis screening and to promote Hepatitis B vaccination.

The present study has the limitations. First, the online questionnaire, which was used in this study was not pretested for reliability and internal consistency. Second, the study was not conducted as a controlled study or an intervention trial. Therefore, improvements in knowledge scores and behavioral changes due to learning the booklets are unknown. Large-scale case-control studies are needed to confirm these results in the future. Third, future studies should consider offering rewards for completing surveys to increase the level of participation.

## Conclusions

In this study, hepatitis education booklets were distributed to all DHWs in the Oita Prefecture in Japan to teach them about hepatitis and its prevention. The booklet was effective in increasing their knowledge about Hepatitis B and C, awareness about the appropriate response to percutaneous injury, and motivation to get tested for the viruses. The use of educational materials can be an excellent means of disseminating correct knowledge of hepatitis to DHWs. In the future, we aim to use this educational booklet to spread awareness about hepatitis to areas other than the Oita Prefecture.

## Appendices

**Table 4:** Supplementary table, comments, and opinions corresponding to Q13 in Table 1, optional HBV: Hepatitis B virus; HCV: Hepatitis C virus

No.	Occupation	Age	Comments and opinions after reading the booklet (optional answers)
1	Dentist	30s	In the booklet, there was a workflow for when a needle stick accident occurs, which was helpful. In the past, I had only cleaned needles after a needle stick, so I felt that regular inspections were necessary.
2	Dentist	30s	It is necessary to secure a partner hospital in case of a needlestick accident at a dental clinic.
3	Dentist	30s	After reading the booklet, I immediately took additional tests for Hepatitis B and C at a recent medical checkup.
4	Dentist	40s	The way to deal with needlestick accidents was clarified, and I would like to make use of it in the future. I am glad that I was able to inform the staff of our hospital about it.
5	Dentist	40s	I decided to test for Hepatitis B and C as soon as possible.
6	Dentist	40s	Dentists have some knowledge about hepatitis and are likely to receive the Hepatitis B vaccine, but non-dentist staff are reluctant to receive the Hepatitis B vaccine because they don't have much knowledge about it or understand its risks. This booklet was easy for non-dentist staff to understand. I was hoping that this would be a good opportunity for the staff to make efforts for prevention.
7	Dentist	40s	I was able to gain knowledge about hepatitis and what to do in case of needlestick injuries. I will make sure that our hospital staff is aware of the needlestick accident.
8	Dentist	50s	I was not aware of the relationship between hepatitis C and lichen planus, so I had not been aware of referring patients with lichen planus to a hepatologist.
9	Dentist	50s	Reading the booklet, I once again understood the importance of hepatitis prevention. Some dental patients do not self-report that they are HBV or HCV carriers, so I was reminded of the importance of standard precautions.
10	Dentist	50s	It was good to be able to confirm our current awareness of infection control. I will share it with the staff in the hospital.
11	Dentist	50s	I would like to know about infection control measures for hepatitis in dental clinics.
12	Dentist	50s	The booklet was easy to understand and helped me to better understand hepatitis.
13	Dentist	50s	I want many people to have a correct understanding of the hepatitis virus and recognize the importance of informing medical professionals about the disease. I also hope that we in the dental profession will have a correct understanding of hepatitis and establish a system to properly provide medical care for infected people, thereby building better relationships between patients and medical professionals.
14	Dentist	50s	I recognized and understood the treatment for Hepatitis C and the relationship between Hepatitis C and lichen planus.
15	Dentist	50s	It was very informative. I would like to use the booklet for staff training.
61	Dentist	50s	So there has been progress in the treatment of Hepatitis C.
17	Dentist	50s	It's easy to understand.
18	Dentist	50s	I have been interested in hepatitis and infection control for a long time and have studied it to a certain extent, but this booklet has deepened my understanding of it again.
19	Dentist	50s	I learned a lot.
20	Dentist	50s	I thought further education on hepatitis was necessary.
21	Dentist	60s	The booklet is helpful.
22	Dentist	60s	The booklet was easy to understand, even for those with limited expertise in hepatitis.
23	Dentist	60s	My previously vague knowledge of hepatitis has been clarified.
24	Dentist	60s	I think it is good to have a better understanding of hepatitis.
25	Dentist	60s	It is concise and easy to understand.
26	Dentist	60s	I had a hard time with Hepatitis B 30 years ago, so I am more concerned about Hepatitis C than others.
27	Dentist	60s	I think it is good to have a better understanding of hepatitis.
28	Dentist	60s	I appreciate the knowledge you provide about hepatitis. Thank you very much.
29	Dentist	60s	The treatment for hepatitis has come a long way, hasn't it?
30	Dentist	60s	It was very helpful.
31	Dentist	70s	I thought the booklet was edited to be easy to understand.
32	Dentist	70s	I'm going to be more careful about nosocomial infections.
33	Dentist	70s	I decided to get the Hepatitis B vaccination.
34	Non-dentist	20s	I was able to learn more about hepatitis.
35	Non-dentist	20s	The content on the difference between Hepatitis B and C was easy to understand.
36	Non-dentist	20s	I thought it was important to get the Hepatitis B vaccine.
37	Non-dentist	20s	Once again, I decided to be careful about needle stick accidents.
38	Non-dentist	20s	I thought it was important to get the Hepatitis B vaccine.
39	Non-dentist	20s	Reading the booklet, I was surprised to learn that the percentage of patients who do not self-report Hepatitis B or C at the dental clinic is quite high. I was reminded of the importance of standard precautions
40	Non-dentist	30s	I understand it well.
41	Non-dentist	30s	There were things I didn't understand in the book, so I learned a lot.
42	Non-dentist	30s	I wanted to know more about it.
43	Non-dentist	40s	After reading the booklet, I now know how to deal with needle stick accidents. Also, I would like to get the Hepatitis B vaccine in the future.
44	Non-dentist	40s	I will be careful not to cause any needlestick accidents in my daily practice.
45	Non-dentist	40s	I felt that I had to understand and deal with hepatitis correctly. However, there are limits to sterilization and disinfection in different dental clinics, and we have no choice but to trust the patients' self-reports. I think not only medical professionals but also the general public need to understand hepatitis better.
46	Non-dentist	40s	It was very informative and I would like to consider getting the Hepatitis B vaccination.
47	Non-dentist	50s	We will address infection control again in our daily work.
48	Non-dentist	50s	I was able to reconfirm what to do after a needlestick accident.
49	Non-dentist	50s	If we don't get dentists to have more risk management, there is a limit to how much infection control we can do from the employee's perspective alone.
50	Non-dentist	50s	Once again, I learned a lot.
51	Non-dentist	50s	It was good to be reminded about hepatitis.
52	Non-dentist	50s	The book was easy to understand, with detailed information about hepatitis and prevention. I have concerns about hepatitis B vaccination because of my allergies, but I would like to consider getting vaccinated.
53	Non-dentist	50s	I learned a lot about hepatitis by reacquainting myself with it.
54	Non-dentist	50s	I thought I was somewhat aware of the dangers of Hepatitis B and C, but I realized that I needed to be even more careful to protect myself.
